# Non-clostridial gas gangrene in a patient with poorly controlled type 2 diabetes mellitus on hemodialysis

**DOI:** 10.1007/s00592-017-1038-2

**Published:** 2017-09-05

**Authors:** Ryo Shigemoto, Takatoshi Anno, Fumiko Kawasaki, Shintaro Irie, Masayuki Yamamoto, Shintaro Tokuoka, Hideaki Kaneto, Kohei Kaku, Niro Okimoto

**Affiliations:** 1 Department of General Internal Medicine 1, Kawasaki Medical School, 2-6-1 Nakasange, Kita-ku, Okayama, 700-8505 Japan; 2 Department of Plastic and Reconstructive Surgery, Kawasaki Medical School, Okayama, 700-8505 Japan; 3 Department of Diabetes, Metabolism and Endocrinology, Kawasaki Medical School, Kurashiki, 701-0192 Japan

Dear Editor,

Gangrene is sometimes observed in patients with type 2 diabetes mellitus (T2DM) and hemodialysis, but in most cases such gangrene is dry and black due to arterial calcification and impaired blood flow. Patients with T2DM are also susceptible to infection. The frequency of non-clostridial gas gangrene is pretty low compared to clostridial one, but once non-clostridial gas gangrene is developed, it leads to high mortality [1–3]. And it is usually caused by gram-negative rods, gram-positive cocci or a combination of microorganisms rather than the clostridium family [4].

**Fig. 1:**
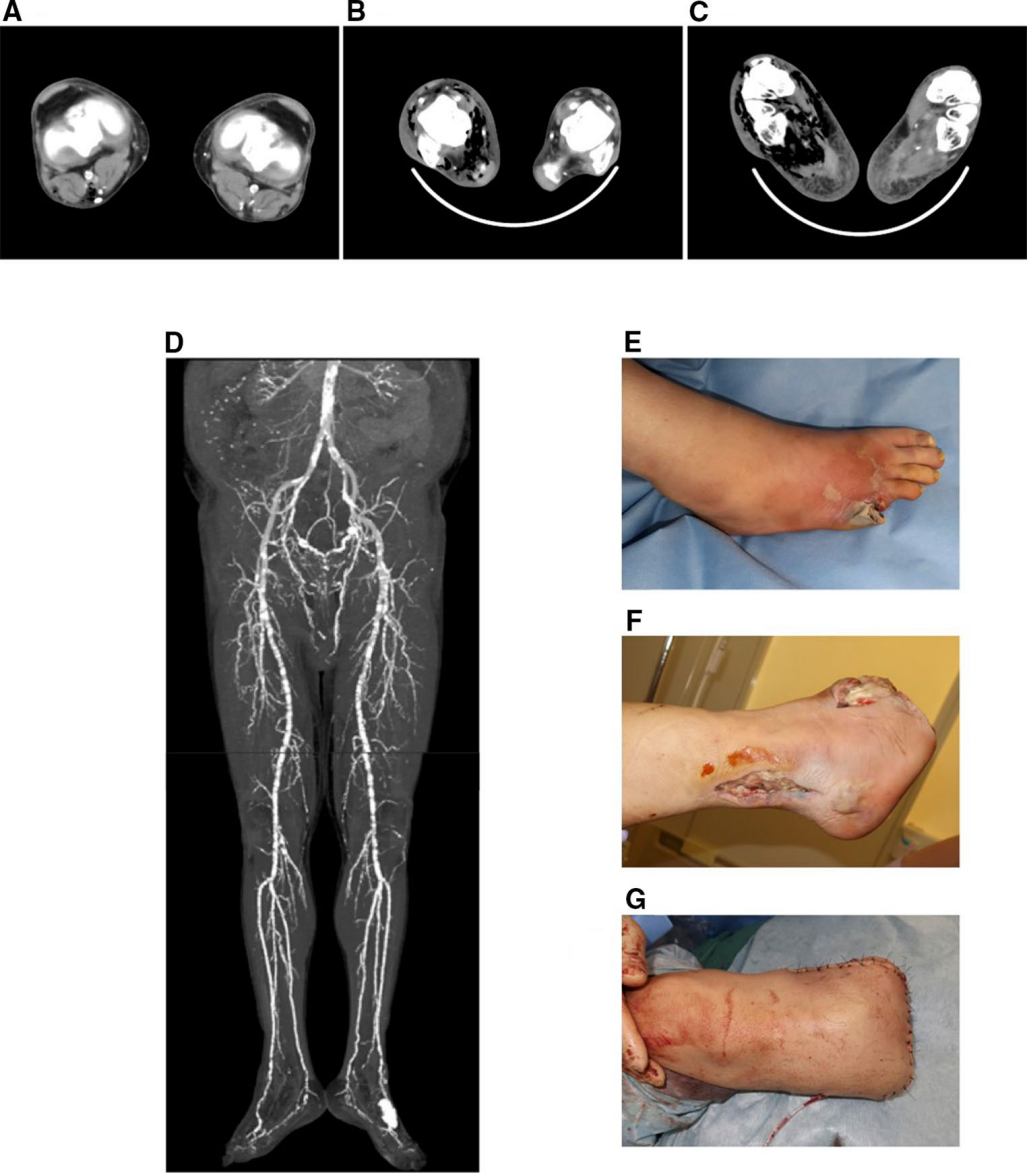
**a**–**c** Leg computed tomography (CT) showing subcutaneous extensive foci of gas throughout his whole leg region. **a** Knee joint region, **b** lower leg region, **c** toe region, **d** CT angiography showing severe arterial calcification throughout the whole body, **e**–**g** non-clostridial gas gangrene in right foot, **e** after amputation of his right little toe, **f** after amputation of his right foot from Lisfranc’s joint, **g** after amputation of his right foot at upper 15 cm of medial condyle

A 52-year-old man with 28-year history of T2DM and receiving hemodialysis was referred to our institution about diabetic gangrene in right little toe. His sensibility was severely disturbed even with pregabalin treatment (100 mg/day). Three weeks before, he felt pain in right little toe, and color of gangrene in right little toe changed to brown or purplish blue. Then, his foot color changed to purplish blue from right toe to right Lisfranc’s joint. He was diagnosed with T2DM at age 25; however, he was hesitated to receive and his lifestyle was very poor. Even after he started therapy for insulin at age 31, he was repeatedly hesitated to treat with T2DM. His details of neuropathy were unknown; however, he had a lot of risk factors for impaired blood flow including not only diabetes and hemodialysis but also hypertension, dyslipidemia, hyperuricemia and smoking. He had diabetic retinopathy at age 39 and had undergone left vitreous surgery at 40 and right vitreous surgery at 47. When he had myocardial infarction and percutaneous transluminal coronary angioplasty at age 50, he started taking aspirin (100 mg/day). His vital signs were as follows: heart rate 89 beats/min, blood pressure 84/45 mmHg and temperature 36.3 °C. Laboratory data were: white blood cell count, 15940/μL (neutrophil 92.0%); red blood cell count, 425 × 10^4^/μL; hemoglobin, 12.1 g/dL; platelet, 15.0/μL; C-reactive protein (CRP), 47.69 mg/dl; procalcitonin, 31.9 ng/mL; creatinine, 10.62 mg/dL; blood urea nitrogen, 53 mg/dL; sodium, 129 mmol/L; potassium, 4.4 mmol/L; chloride, 96 mmol/L; plasma glucose, 484 mg/dL; hemoglobin A1c (HbA1c), 9.7%; glycoalbumin 29.2%. Computed tomography (CT) revealed subcutaneous extensive foci of gas throughout his leg from the right tip of the toe region to the knee joint region (Fig. 1a–c). Furthermore, CT angiography revealed severe arterial calcification throughout the whole body (Fig. 1d). Peripheral sensory neuropathy of his remained foot was severe, and he could not feel pain on his foot. His kneecap reflex and Achilles tendon reflex were lost. He could feel vibration on foot for only 2 s. Monofilament test revealed that he could feel the 5.07 (10 g) filament on the beginning part of big toe, but not another site. Nerve conduction study revealed that compound muscle action potential (CMAP) was low and sensory nerve action potential (SNAP) was not detected on his remained foot. These results suggest that he had severe diabetic neuropathy.

First, we performed the debridement and intravenous antibiotics therapy (3.0 mg/day of meropenem hydrate). *Arcanobacterium haemolytic*, *group G β*-*streptococcus*, *peptostreptococcus sp.*, *prevotella intermedia/disiens* and *gram*-*negative anaerobic bacteria* were identified in culture from odoriferous pas with wound on admission and on next day. No bacteria were found in blood. Therefore, we finally diagnosed him as non-clostridial gas gangrene. His right foot infection was not improved with continuous debridement for 3 day, because his right foot was fully with necrotic tissue and pass under skin when we performed the debridement. Therefore, we amputated his right foot from Lisfranc’s joint three days after admission (Fig. 1e–f). Since three weeks later his laboratory data were not normalized (WBC, 6500/μL (neutrophil 72.0%); CRP, 5.92 mg/dl)) with continuous debridement, we finally amputated his right foot at upper 15 cm of medial condyle (Fig. 1g). Then, his general condition was improved and his infection markers were almost normalized (WBC, 6510/μL (neutrophil 47.8%); CRP, 0.86 mg/dl)) 1 week after amputation. We stopped 3.0 mg/day of meropenem hydrate for total 1 month. His glycemic control was good; fasting plasma glucose was under 100 mg/dL with bolus insulin (4 units of aspart before each meal).

In many cases, gangrene in subjects with diabetes and receiving hemodialysis is dry and black gangrene due to impaired blood flow. In contrast, the development of non-clostridial gas gangrene is very rare in such subjects. In this report, however, we showed a case with T2DM and hemodialysis who developed non-clostridial gas gangrene, but not gas gangrene. In addition, since very severe arterial calcification was observed throughout the whole body (Fig. 1d), it seemed that the possibility of dry and black gangrene was higher rather than of gas gangrene. Nonetheless, non-clostridial gas gangrene was developed in this subject. Since the frequency of non-clostridial gas gangrene in subjects with diabetes and hemodialysis is very low, it remains unknown which factors and/or conditions are involved in the development of non-clostridial gas gangrene. To address this point, it would be necessary to perform further analysis with a substantial number of subjects.

Taken together, although it has been paying much attention to the onset of dry and black gangrene in subjects with diabetes and hemodialysis, we should keep in mind the possibility that non-clostridial gas gangrene is also developed in such subjects.
